# Determinants of Thoroughbred yearling sales price in the UK

**DOI:** 10.1002/vro2.81

**Published:** 2024-06-09

**Authors:** Rebecca R. Mouncey, Pablo Alarcon, Kristien L. Verheyen

**Affiliations:** ^1^ Department of Pathobiology and Population Sciences The Royal Veterinary College Hatfield UK

## Abstract

**Background:**

Industry‐level figures suggest that up to two‐thirds of Thoroughbred breeding operations in the UK are unprofitable and that around half of sales transactions of Thoroughbred yearlings, commercial breeders’ predominant income source, return a loss. The industry strategy currently endorses investment in stallion covering fee; however, to date, a comprehensive evaluation of sales price determinants in the UK setting is lacking and could better inform economic decision making to improve profitability.

**Methods:**

Sales catalogue and Weatherbys’ stud book data from all Thoroughbred yearlings sold at the 2020 Tattersalls October yearling sale in the UK were used to build a hedonic sales price model. Explanatory variables representing sire, dam, yearling and sales attributes were evaluated. The final model's accuracy was assessed using out‐of‐sample data from all yearlings sold in the equivalent 2021 sale.

**Results:**

In 2020, a total of 1506 catalogued yearlings, representing around 30% of the UK Thoroughbred foal crop, were sold, with a median price of £42,575 (interquartile range 15,750‒105,000; range 840‒3,570,000). The sires’ covering fee, maternal siblings’ race performance attributes, whether the yearling was the dams’ first foal, consignment size, catalogue book and day of sale within book significantly influenced auction price; however, relationships were complex with significant interaction and confounding observed. The mean model forecasting error was £2074. The use of data from only one sale could affect generalisability.

**Conclusions:**

These novel findings can inform breeding decisions to maximise profitability, give context for current industry strategies and can inform valuations of breeding stock.

## INTRODUCTION

The UK's Thoroughbred breeding industry is estimated to contribute more than £375 million in gross value added to the UK economy and, directly and indirectly, support more than 20,000 jobs.^1^ Despite this, the breeding industry currently faces important economic challenges due to declining profitability and loss of breeders, which threatens its sustainability.^1^, ^2^, ^3^ The latest industry‐level report showed that from 2009 to 2021, the number of breeders (registered broodmare owners) dropped by 35%, from 4621 to 3017, reducing the UK's broodmare band and foal crops by 23% (2452 mares and 1313 foals, respectively).^3^ The report suggested that due to cost pressures, which were estimated to have increased annually by 4% since 2013, the majority of breeders were now unprofitable, with the average yearling sale, an important source of breeder income,^3^, ^4^ resulting in a loss of more than £30,000 in 2021.^3^ The report projected that, if breeders continued to leave the industry at present rates due to declining profitability, by 2050, the UK foal crop would reduce by one‐third, threatening the sustainability of the UK racing industry, which is ‘overwhelmingly reliant on the UK's production of quality bloodstock’.^3^

The yearling sales market is polarised, with the mean profit supported by a small number of extremely profitable sales from a small number of breeders, while around two‐thirds of transactions are estimated to be unprofitable.^3^ Industry‐level figures suggest a shift towards the use of more expensive stallions, with stallion covering fee (the fee paid by the mare owner for her to be bred with the sire) averaging just more than £20,000 in the UK in 2021.^3^ Industry‐level analyses, which calculated the average profit per yearling sale by bracket of stallion covering fee, suggested that the ‘break‐even point’ for breeders in 2021 was for stallion covering fees of £175,000 or above.^3^ Due to the lengthy production cycle of around 30 months from covering the mare by the stallion to the point of sale of the yearling, such fees represent an important investment and cost to carry by breeders. Current industry strategy endorses investment in stallion covering fee to increase returns^3^; however, although industry‐level analyses provide important benchmarking in this regard, a more comprehensive microeconomic approach is required to further understand specific relationships before the potential merits of any such investments can be evaluated.

Hedonic price modelling fits an ordinary least squares regression model to sales data to estimate the contribution of various characteristics or attributes to an item's sales price. This method is commonly utilised to understand determinants of price by allowing for the examination of how each attribute uniquely contributes to the overall value of the items while simultaneously accounting for the effects of other attributes.^5^ Hedonic price modelling has been utilised to evaluate determinants of yearling sales price in Australia,^6^, ^7^ the USA^8^, ^9^ and New Zealand.^10^ To the best of the authors’ knowledge, there has been only one previous UK study utilising figures from almost two decades ago.^11^ Given the current economic climate, furthering understanding of determinants of Thoroughbred yearling price, particularly the role of stallion covering fee, is vital to help inform economic decision making. Up‐to‐date analyses could not only help to inform breeding strategies to maximise profitability but, by evaluating models’ forecasting accuracy, the findings could potentially also provide useful additional information for breeders in terms of expected auction prices and aid in the valuation of stock.

Therefore, the objectives of the present study were to use hedonic modelling of sales and stud book data to (1) investigate determinants of UK Thoroughbred yearling sale price, (2) evaluate the role of the stallion covering fee and (3) evaluate determinants’ price forecasting ability.

## METHODS

### Data collection

Sales catalogue data were collected for the 2020 and 2021 Tattersalls October yearling sales from www.tattersalls.com/sales. The following data were retrieved for all catalogued lots: yearling's date of birth, the book in which it was catalogued and the day of sale within the book, the dam, whether it was the first foal (FF) from the dam, whether either the dam or at least one maternal sibling had won a race (DRW or SRW) or won a black type race (DBT or SBT; first, second and third in a group/graded or listed stakes race as approved by the Cataloguing Standards Guide), the sire, the vendor, the purchaser and the sales price. The sires’ advertised covering fee for 2018 (the year of covering for 2019 born yearlings sold in 2020) and 2019 (for 2020 born yearlings sold in 2021), along with whether the stallion was a first season sire (FSS) in that season, were collected where available from www.racingpostbloodstock.com. The number of stallions covered by registered Weatherbys mares was collected from Weatherbys return of mares for the respective seasons (2018 and 2019).^3^

### Data processing

The data were imported into Stata (Release 16, StataCorp) and sire, studbook and sale catalogue data were merged by sire name. All covering fees were converted to GBP at rates of exchange of 1 January of the season of interest (2018: 0.89 EUR = 1 GBP, 0.78 USD = 1 GBP and 2019: 0.90 EUR = 1 GBP, 0.78 USD = 1 GBP). The sales price was converted from guineas to GBP (1.05 guineas = 1 GBP). The yearlings’ age (in days) at the time of sale was calculated as the date of sale minus the date of birth; the number of lots consigned by the vendor was calculated as the total number of lots consigned by the vendor across the whole sale in the respective year.

### Data analyses

Only sold lots were included in analyses; lots that were withdrawn, those that passed through the ring unsold or sold privately were therefore excluded. Lots that were recorded as having been bought by the vendor (that is, bought‐in) were also excluded because such prices may not be a true representation of the prevailing market value.^11^ The 2020 sales dataset was used as the study sample (to construct the model) and the 2021 sales dataset was reserved to conduct out‐of‐sample forecasting of the final model.

Histograms were plotted and visually inspected for normality. The data were described using the mean, standard deviation and range if normally distributed, and median, interquartile range (IQR) and range if non‐normally distributed.

Attributes (Table 1) were fitted as fixed effects to build a hedonic model of sales price. Due to the possibility of heteroskedasticity in the error term, the model was fitted using robust standard errors. The functional form of continuous variables was specified using Cox‒Pesaran‒Deston and *J*‐tests, resulting in the following log‐linear model being fitted.

$$\ln\left(y_{i}\right)=\alpha +\beta_{\mathrm{Sire}}+\beta_{\mathrm{Dam}}+\beta_{\mathrm{Yearling}}+\beta_{\mathrm{Sale}}+\varepsilon_{i}$$

where the dependent variable is the natural logarithm of the sales price of a yearling and the price is a function of various attributes of the sire, dam, yearling and sale.

**TABLE 1:** Definitions and expected signs of all variables utilised to construct the hedonic price model to estimate Thoroughbred yearling sales price.

Variable	Expected sign	Definition of variable
Dependent variable		
ln sales price	n/a	The natural logarithm of the hammer price for the yearling in GBP (1 GBP = 1.05 guineas)
Sire attributes		
ln stud fee	+	The natural logarithm of the advertised covering fee of the yearling's sire in GBP
First Season Sire	+	FSS = 1 if the stallion was a first season sire and 0 otherwise
Coverings	+	Number of mares covered by the stallion i, as per Weatherby's stud book return of mares
Dam attributes		
Dam Race Winner	+	DRW = 1 if dam won at least one race and 0 otherwise
Dam Black Type	+	DBT = 1 if dam won black type^a^ and 0 otherwise
Sibling Race Winner	+	SRW = 1 if at least one maternal sibling won at least one race and 0 otherwise
Sibling Black Type	+	SBT = 1 if at least one maternal sibling won black type^a^ and 0 otherwise
First Foal	‒	FF = 1 if the first foal born out of the dam and 0 otherwise
Yearling attributes		
Colt	+	Colt = 1 if the yearling is an uncastrated male and 0 otherwise (female or castrated)
Age	+	Age of the yearling in days at the time of sale (passing through ring)
Sale attributes		
Lots vendor	+	Total number of lots consigned by the vendor over the whole sale (i.e., consignment size)
Book 1, 2, 3 or 4	‒	Categorical variables representing the yearling's placement in the sale by book; the reference variable is book 1.
Day 1, 2 or 3	‒	Categorical variable representing the day of the yearling's sale within the book; the reference variable is day 1

Abbreviation: ln, natural logarithm.

^a^ First, second or third in a group/graded or listed stakes race as approved by the Cataloguing Standards Guide.

**FIGURE 1:**
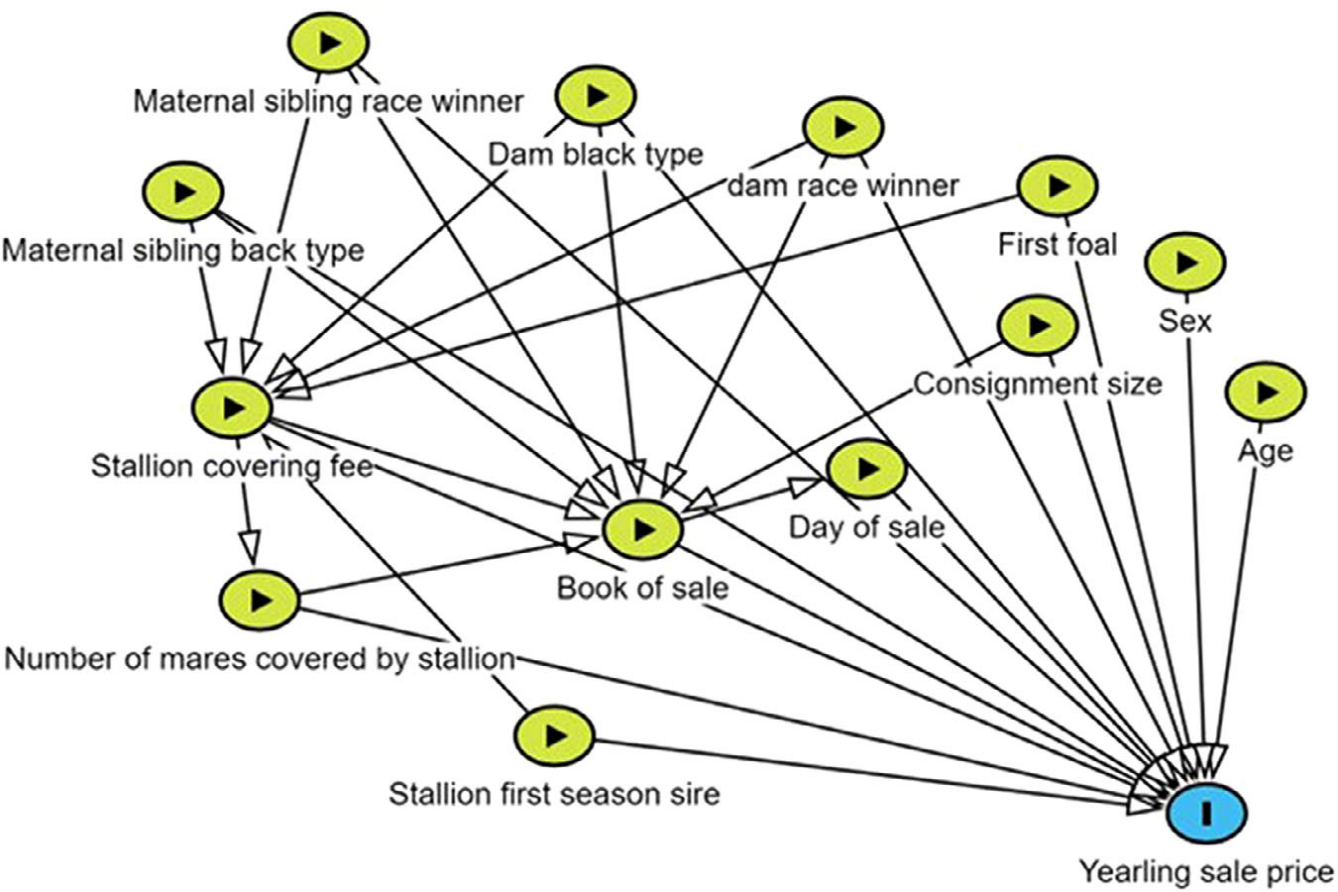
A directed acyclic graph of potential relationships between variables utilised to inform a hedonic price model to estimate Thoroughbred yearling sales price.

A directed acyclic graph (DAG) of all variables^12^ was constructed a priori to inform analyses (Figure 1). The DAG highlighted potential for both interaction and confounding between explanatory variables. To evaluate confounding factors, the final model was constructed by stepwise inclusion of categories of explanatory variables (sire, dam, yearling and sale attributes; Table 1). There was deemed to be evidence of confounding if coefficient estimates changed by more than 20% when further categories of explanatory variables were added to the model. Interactions between all predictor variables were tested. There was deemed to be evidence of interaction if the likelihood ratio test (LRT) comparing a model with the interaction term to a model without resulting in *p*‐value of less than 0.05, and the interaction term was retained in the final model as appropriate.

To evaluate fit of the final model, a Link test was performed to test model specification, a Whites test was performed to test for heteroscedasticity and graphs were plotted to check the assumptions of normality of residuals and linearity between predictors and outcome.

Marginal values (in GBP) across the sample data (using the *margins* command in Stata^13^) were calculated to estimate either the effect of a one‐unit increase in a continuous independent variable or the difference between the presence and absence of a categorical independent variable on sales price for all significantly associated variables in the final model (*p* < 0.05). The potential bias of reverse transformation from ln GBP to GBP^14^ was accounted for by including a function of the variance of errors in all predictions as follows:

$$E\left(Y|X\right)=e^{x\beta }e^{\sigma 2/2}$$

where *σ* ^2^ is the variance of the errors, which was estimated using Stata's *gsem* command^15^ syntax to run the final linear model, which includes the error variance in the model output.

Plots of predicted mean sales prices (*margins plots* ^13^) were generated as required to further understand any interactions.

The 2021 dataset was used to test the forecasting ability of the final model. The estimated coefficients were applied to the out‐of‐sample observations and the sales price predicted by the model was compared to the actual sales price recorded for the yearling to give the forecasting error (the true sales price minus the predicted sales price as estimated by the model). This was first evaluated on the log scale, as would appear to be convention for testing forecasting ability of log‐linear models.^6^ We also tested forecasting ability of the transformed estimates, which were calculated using the variance of error adjustment to minimise retransformation bias as described above. From the forecasting error the mean squared forecast error (MSE) or the risk function, the mean absolute error (MAE) and root mean squared error (RMSE), all of which measure the spread of forecasting errors, and the mean absolute percentage error, which indicates how large the forecasting error is as a percentage of the actual value, thus expressing accuracy as a percentage, were calculated. Because we were comparing models with different scales (log‐transformed and exponentiated predictions), the normalised RMSE (nRMSE) was calculated by dividing the RMSE by the range of *y*.

## RESULTS

A total of 2048 lots were catalogued for sale in 2020 and 2019 in 2021, representing 42% (2048/4816) and 44% (2019/4539) of the 2019 and 2020 UK foal crops, respectively.^16^ A summary of the total numbers of lots catalogued, lots withdrawn, lots passed through the ring, and lots not sold and sold for the 2020 and 2021 Tattersalls October yearling sales is presented in Supporting Information S1. Overall clearance rates were high, with less than 3% of lots not sold (46/2048 in 2020 and 53/2019 in 2021).

The study sample consisted of *n* = 1506 lots sold (excluding lots bought‐in) in 2020 and the test set consisted of *n* = 1542 lots sold (excluding lots bought‐in) in 2021. The median sale price for the study sample was £42,575 (IQR 15,750‒105,000; range 840–3,570,000) and the median stallion covering fee was £20,000 (IQR 10,680‒35,600; range 2670‒450,000). The distributions of stallion covering fee and sale price by catalogue book are given in Table 2.

**TABLE 2:** Distribution of stallion covering fee and sale price by catalogue book number for 1506 yearling Thoroughbreds sold (excluding buy‐ins) at the 2020 Tattersalls October yearling sale.

	Stallion covering fee (GBP)			Sale price (GBP)		
	Median	Interquartile range	Range	Median	Interquartile range	Range
Book 1	40,000	26,700‒75,600	5340‒450,000	126,000	73,500‒220,500	10,500‒3,570,000
Book 2	26,700	13,350‒35,600	4450‒175,000	52,500	29,400‒91,350	3150‒708,750
Book 3	10,680	7000‒15,000	2000‒120,150	10,762	5512‒22,050	840‒136,500
Book 4	6000	4500‒8000	3000‒17,800	3150	1260‒6300	840‒12,600
All	22,250	10,680‒35,600	2000‒450,000	42,000	15,750‒99,750	840‒3,570,000

Further information on Tattersalls sales policies, stallion covering fees and Weatherbys’ stud book is provided in Supporting Information S2. The cataloguing distribution (lots by book and day of sale) of sold lots (excluding buy‐ins) for the study sample is provided in Supporting Information S3. Descriptive statistics of all variables used to build the hedonic price model and descriptive statistics of the out‐of‐sample test set (*n* = 1542 lots sold in 2021) are provided in Supporting Information S4 and S5, respectively.

The coefficients following the addition of each group of attributes during model building, the final model and the average marginal values across the sample for significantly associated (*p* < 0.05) determinants in GBP are given in Table 3. In the final model, stallion covering fee, sibling race performance attributes (SRW and SBT) being a FF from the dam and being a colt were all positively associated with sales price. For example, if all other attributes were equal, a colt was estimated to sell for 10% (or on average across the sale around £10,000) more than a filly (coefficient 0.10, marginal value colt £10,141.06; Table 3). Conversely, consignment size (the total number of lots consigned by the vendor over the sale) was negatively associated with sales price. For example, if all other attributes were equal, a yearling from a consignor with 10 lots was estimated to sell for 0.3% less (or on average across the sale around ‒£300) than a yearling from a consignor with nine lots (coefficient −0.003, marginal value ‒£295.76; Table 3).

**TABLE 3:** Coefficients and standard errors (SEs) following the addition of each group of attributes during model building and the final multivariable model with average marginal values (GBP) across the sample for variables significantly associated with yearling sales price in the final model.

	Model building				Final model						
Variable	Coefficient (SE)	Coefficient (SE)	Coefficient (SE)	Coefficient (SE)	Coefficient (SE)	95% confidence interval		*p*‐Value	Average marginal value^a^	95% confidence interval	
Sire attributes											
ln StudFee	0.88 (0.04)^*^	0.82 (0.04)^*^	0.82 (0.04)^*^	0.27 (0.04)^*^	**0.50 (0.06)** ^*^	0.38	0.62	<0.0001	29,645.23	20,792.68	38,497.79
FSS	0.17 (0.08)^*^	0.15 (0.08)^*^	0.15 (0.07)^*^	‒0.09 (0.06)	‒**0.11 (0.06)**	‒0.23	‒0.01	0.08			
Coverings	0.0009 (0.0006)	0.0005 (0.0006)	0.0007 (0.0006)	0.0005 (0.0005)	**0.0005 (0.0005)**	‒0.0004	0.001	0.29			
Dam attributes											
DRW		‒0.07 (0.07)	‒0.06 (0.06)	0.001 (0.05)	**0.01 (0.05)**	‒0.11	0.09	0.89			
DBT		0.24 (0.08)^*^	0.24 (0.07)^*^	0.13 (0.06)^*^	**0.08 (0.06)**	‒0.04	0.20	0.20			
SRW		0.14 (0.08)	0.15 (0.08)	0.14 (0.06)^*^	**1.49 (0.52)** ^*^	0.46	2.52	0.005	42,919.58	‒9987.46	18,426.63
SBT		0.54 (0.08)^*^	0.55 (0.08)^*^	0.19 (0.06)^*^	**0.18 (0.06)** ^*^	0.06	0.31	0.004	18,287.29	5316.816	31,258.41
FF		0.41 (0.09)^*^	0.36 (0.09)^*^	0.19 (0.07)^*^	**0.18 (0.07)** ^*^	0.04	0.32	0.01	18,355.46	2935.04	33,775.88
Yearling attributes											
Colt			0.33 (0.06)^*^	0.14 (0.06)^*^	**0.10 (0.05)** ^*^	0.01	0.19	0.02	10,141.06	1491.68	18.790.43
Age			0.002 (0.001)^*^	0.001 (0.0008)	**0.001 (0.008)**	‒0.0007	0.002	0.26			
Sale attributes											
Lots Vendor				‒0.003 (0.001)^*^	‒**0.003 (0.001)** ^*^	‒0.006	‒0.0002	0.04	‒295.76	‒572.42	‒19.10
Book 2				‒0.76 (0.06)^*^	**2.25 (0.072)** ^*^	0.82	3.66	0.002	‒81,748.49	‒96,533.73	‒66,962.40
Book 3				‒2.14 (0.08)^*^	**1.22 (0.085)**	‒0.45	2.89	0.15			
Book 4				‒3.34 (0.15)^*^	‒**1.75 (2.15)**	‒5.98	2.47	0.42			
Day 2				‒0.24 (0.05)^*^	‒**0.60 (0.01)**	‒0.26	0.13	0.55			
Day 3				‒0.11 (0.06)	**0.13 (0.01)**	‒0.06	0.32	0.42			
ln StudFee # book 2					‒**0.29 (0.07)** ^*^	‒0.42	‒0.15	<0.0001			
ln StudFee # book 3					‒**0.30 (0.08)** ^*^	‒0.46	‒0.13	<0.0001			
ln StudFee # book 4					‒**0.13 (0.024)**	‒0.61	0.34	0.58			
ln StudFee # SRW					‒**0.13 (0.05)** ^*^	‒0.23	‒0.31	0.01			
Book 2 # day 2					**0.18 (0.13)**	‒0.07	0.44	0.16			
Book 2 # day 3					‒**0.16 (0.13)**	‒0.42	0.10	0.23			
Book 3 # day 2					‒**0.66 (0.14)** ^*^	‒0.93	‒0.39	<0.0001			
Prob > *F*	<0.0001	<0.0001	<0.0001	<0.0001	**<0.0001**						
*R* ^2^	0.33	0.37	0.38	0.61	**0.63**						
AIC	4773.11	46,990.34	4658.14	3977.46	**3911.13**						
RMSE	1.18	1.14	1.13	0.90	**0.88**						

*Note*: DBT—first, second or third in a group/graded or listed stakes race as approved by the Cataloguing Standards Guide. Akaike information criterion (AIC) is an estimator of prediction error and thereby the relative quality of statistical models for a given set of data. The lower the AIC the better the model‐fit, that is, the better predictive performance.

Abbreviations: DBT, dam black type; DRW, dam race winner; FF, first foal; FSS, first season sire; ln, natural logarithm; RMSE, root mean squared error; SBT, sibling black type; SRW, sibling race winner.

^a^ Average marginal value calculated across the sample.

* *p* < 0.05.

During model building, confounding was observed between stallion covering fee, FSS, DBT, SBT, FF, yearling sex, yearling age and sales attributes (coefficients changed by >20% with the addition of sales attribute variables to the model; Table 3). This suggested that stallion covering fee was not only associated with the yearling's sales price but also, for example, with the yearling's book placement in the catalogue.

**FIGURE 2:**
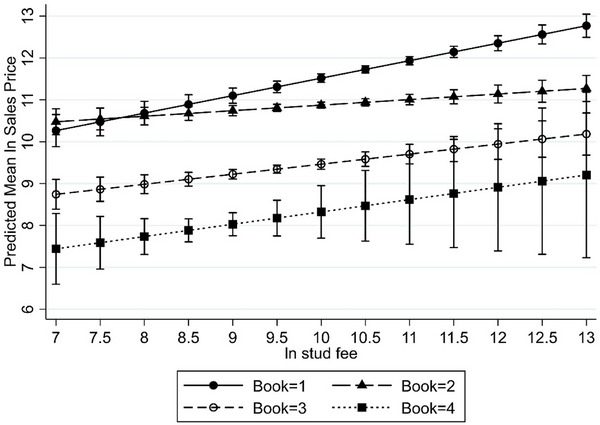
Plot of the predicted mean sales price (natural logarithm (ln) of sales price and 95% confidence interval) by stallion covering fee (natural logarithm [ln]) for Thoroughbred yearlings catalogued in books 1, 2, 3 and 4 of the 2020 Tattersalls October yearling sale.

**FIGURE 3:**
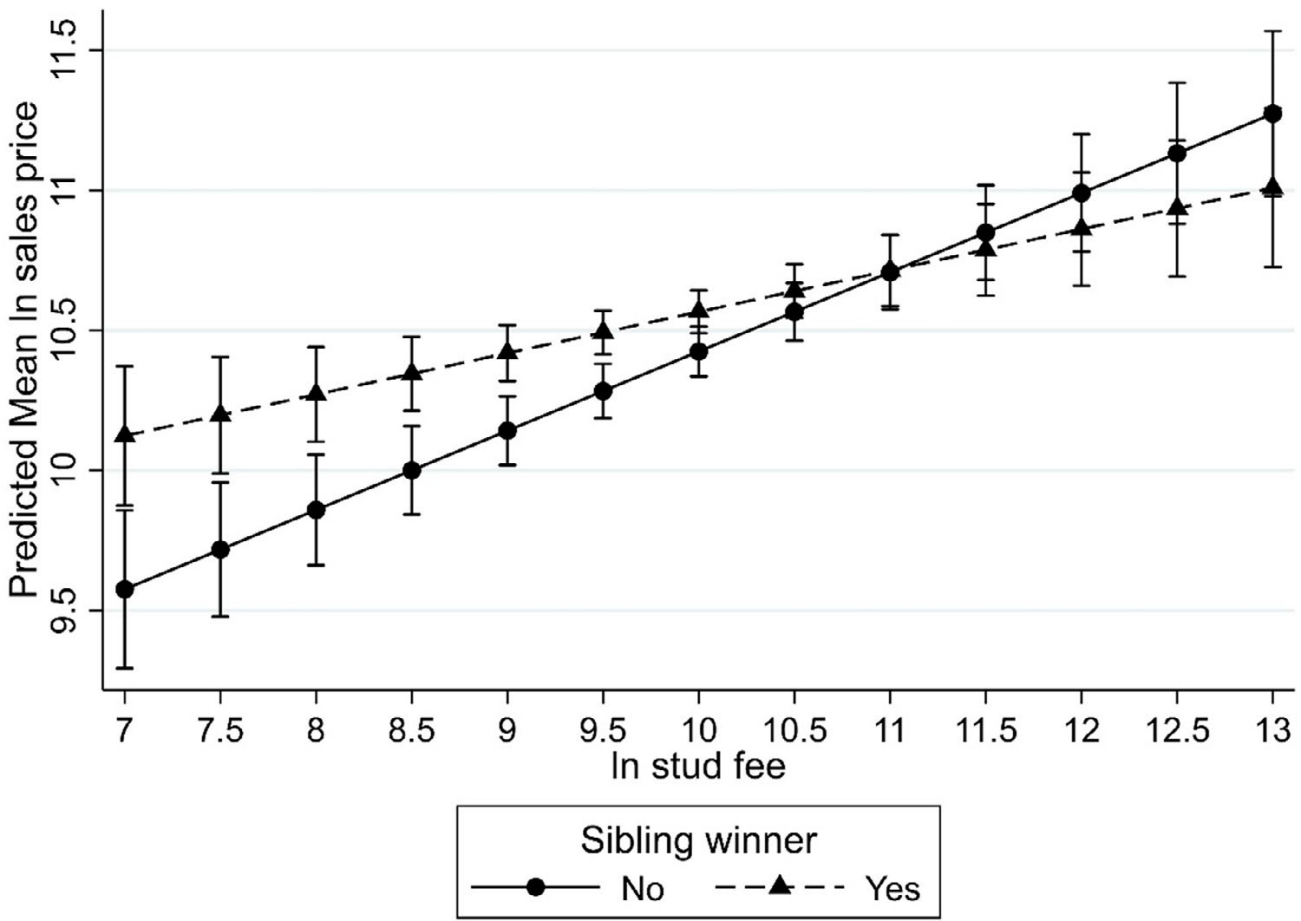
Plot of the predicted mean sales price (natural logarithm [ln] of sales price and 95% confidence interval) by stallion covering fee (ln) for Thoroughbred yearlings sold in the 2020 Tattersalls October yearling sale, with and without at least one maternal sibling who had won at least one race.

**FIGURE 4:**
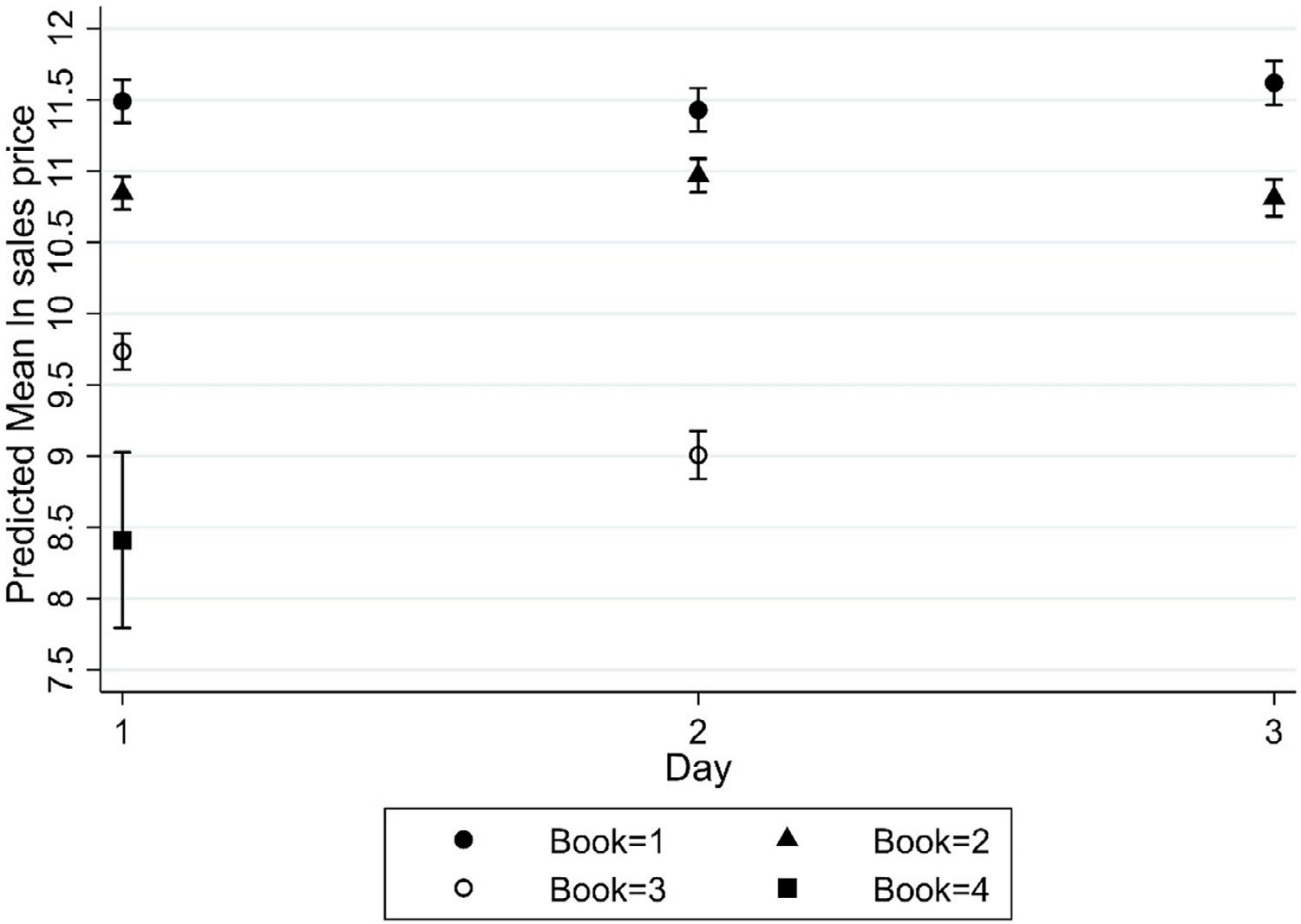
Plot of the predicted mean sales price (natural logarithm (ln) of sales price and 95% confidence interval) by day of sale for Thoroughbred yearlings sold in books 1, 2, 3 and 4 of the 2020 Tattersalls October yearling sale.

To facilitate understanding of interaction in the final model, plots of the predicted margins (predicted mean natural logarithm of sales price) for all interaction terms with 95% confidence interval are given in Figures 2, 3, 4. An interaction was observed between stallion covering fee and book of sale (LRT, *p* = 0.002), which means that, all other attributes being equal, the effect of sire's covering fee on sales price varied, depending on the yearling's catalogue position. Compared to book 1, the effect of increases in stallion covering fee on sales price was significantly smaller in books 2 and 3. Because the stallion covering fee was expressed in the model as a natural logarithm, its parameter value (coefficient; Table 3) represents the elasticity of yearling price. Therefore, in book 1, a 10% increase in investment in stallion covering fee was estimated to result in a 5% increase in return from sales price (coefficient 0.50), whereas in books 2 and 3, a 10% increase in stallion covering fee was estimated to increase sales price by around 3% less (interaction term coefficients −0.30 and −0.29, respectively) than in book 1. At sample means for book 1 (mean stud fee ∼£73,000, mean sales price ∼£235,000), a £1 investment in covering fee therefore returned a £1.60 increase in sales price. However, for books 2 and 3 (book 2 mean stud fee ∼£30,000, mean sales price ∼£80,000; book 3 mean stud fee ∼£14,000, mean sales price ∼£16,800), £1 investment yielded just £0.53 and £0.24, respectively.

Interaction was also observed between stallion covering fee and whether the dam had bred a sibling who had won a race (SRW) (*p* = 0.01), meaning that the effect of the yearling having a race‐winning sibling (SRW) on sales price varied depending on the sire's covering fee. To further understanding, the corresponding interaction plot (Figure 3) demonstrated that, all other attributes being equal, the effect of SRW decreased as covering fee increased; furthermore, the positive effect of this attribute was significant only for yearlings from stallions with covering fees of less than around £13,000 (log‐transformed e^9.5^; Figure 3).

Interaction was also observed between the book and day of the sale within the book (*p* < 0.0001; Table 3), indicating that the effect of book on yearling's sales price varied depending on which day within the book the yearling sold on. All other attributes are equal: yearlings sold on day 2 of book 3 sold for significantly less compared to book 3 yearlings that were sold on day 1 (Figure 4 and Table 3).

The associations between the stallion covering fee, book of sale and sales price in the final model were particularly nuanced. On average, across the sale, yearlings in book 2 sold for around ‒£80,000 less than yearlings in book 1 (marginal value book 2; ‒£81,748.49; Table 3). However, the interaction between stallion covering fee and book of sale suggested that all other attributes being equal; yearlings from stallions with the lowest covering fees (less than around £1800; e^7.5^; Figure 2) were predicted to sell for more in book 2 compared to book 1.

A Link test (*p* = 0.50) suggested no evidence to reject the null hypothesis that the model was specified correctly and a Whites test (*p* = 0.30) suggested no evidence to reject the null hypothesis of constant variance. The residuals were normally distributed with no evidence of heteroscedasticity (see Supporting Information S6) and there was linearity between the predictors and the outcome (see Supporting Information S7).

When applying the final model coefficients to the 2021 data as an out‐of‐sample test set, the model had a pseudo *R* ^2^ = 0.64. The measures of forecasting ability of both the log‐transformed and exponentiated predictions are presented in Table 4. The nRMSE suggested that accuracy may be better at the original scale (lower nRMSE), with a MAE of just over £2000. The mean percentage error was perhaps less favourable at 65%. The means of actual and predicted sales prices were similar, suggesting that the model's forecasting ability around the sample mean was adequate; therefore, the forecasting ability may be less accurate towards the extremes of sample data.

**TABLE 4:** Measures of forecasting ability of the final model for predictions on the log scale and for exponentiated predictions using the 2021 Tattersalls sales data as the out‐of‐sample test set.

Measure	Log‐linear	Exponentiated
Mean squared error	0.71	1.12 × 10^10^
Normalised square root of the mean squared error	0.11	0.07
Mean absolute error	0.25	£2074.30
Mean absolute percentage error	1.79%	65%

## DISCUSSION

This study provides up‐to‐date estimates, together with original and additional understanding of determinants of yearling Thoroughbred sales price in the UK. The novel approach of constructing a DAG prior to model building informed the investigation of both confounding factors and interactions, which in turn highlighted complex, nuanced relationships between price determinants that have not previously been described. Given the economic challenges currently facing the UK Thoroughbred breeding industry,^3^ such enhanced understanding not only provides important additional information to better inform economic decision making but also gives vital context for recently proposed industry strategies.^3^ Alongside this, the evaluation of the final model's forecasting ability, the first of its kind in a UK setting, allows the present findings to be utilised to forecast expected auction prices at this sale and aid in the valuation of stock.

Consistent with previous studies, the stallion covering fee was positively associated with yearling sales price.^6^, ^8^, ^9^, ^11^, ^17^, ^18^, ^19^ In contrast to these previous studies, however, the present work has provided novel and additional information regarding the role of stallion covering fee by investigating interactions and confounding. Catalogue book placement is determined by the sales house, following examination of all yearlings by members of the sales team prior to the sale, with yearlings deemed to be of the highest quality (as judged both on pedigree and physical attributes) and therefore expected to attain the highest prices, being placed in book 1. Therefore, perhaps unsurprisingly, the stallion covering fee influenced both the yearlings’ catalogue placement (book) and sales price (confounding). It was interesting, however, and perhaps less intuitive, that the effect of stallion covering fee on sales price varied depending on the book in which the yearling was catalogued (interaction). This finding is particularly relevant when considered in the context of strategies advocated by the most recent UK breeding industry report,^3^ which were based on initiatives to improve breeders’ accessibility to higher quality and therefore more expensive stallions. The findings from this study suggest that any such investment initiatives may be effective only under certain circumstances. Investments in stallion covering fee for mares with racing and progeny attributes likely to place their offspring in books 2 and 3 should be undertaken with caution, as predicted returns are on average lower than investments (£1 investment returns just £0.53 and £0.24 books 2 and 3, respectively). In contrast, investments in stallion covering fee for mares with strong pedigree and proven progeny whose offspring are likely to be placed in book 1 are perhaps better justified, as returns are on average larger and positive (£1 returns £1.60).

Cataloguing of lots into books or ‘premier’ or ‘select’ sales is commonly undertaken across sales of various ages of Thoroughbreds (weanlings, yearlings, 2‐year‐olds) and across geographical regions. Therefore, the present findings of both confounding and interactions between catalogue book and other sales attributes and sales price not only provide important additional information for the Thoroughbred sales and breeding industry, but also perhaps, bring into question sales companies’ cataloguing processes. Our findings suggest that it could be more equitable if all horses were sold under the same book, allowing the auction itself to determine the true value of each individual, without purchasers being potentially biased by sales companies’ inspections and cataloguing processes.

Unlike book placement, positioning within books, that is, the day of sale, is determined by alphabetical order of the dam's name. Therefore, it was interesting that in book 3 (which comprised 2 days), those selling on the second day sold for significantly less, compared to those selling on the first day. It could be hypothesised that towards the lower end of the sale, demand may decline and/or there may be fewer purchasers present on subsequent days because in lower books, several lots are considered to be similar by buyers, meaning that they tend just to bid on the first one rather than waiting. Such buyer behaviour would therefore disadvantage those lots catalogued on subsequent days and warrants further consideration by sales houses.

In accordance with most previous studies,^7^, ^8^, ^9^, ^11^, ^20^, ^21^ colts were demonstrated to commanded significantly higher sales prices than fillies. Additionally, investigations of confounding revealed that sex was also associated with catalogue placement. Again, given that the elasticity of colts reduced from selling for on average 33% more than fillies to just 14%, when sales attributes (book) were added to the final model (coefficient of colt 0.33 and 0.14, respectively; Table 3), bringing into question cataloguing processes. Parts of the market prefer colts, with some of the most prestigious and lucrative pattern races restricted to them. In addition, colts have been demonstrated to have higher rates of body mass gain compared to fillies between three months and 18 months of age,^22^ suggesting that they may generally be more physically developed at the time of cataloguing inspections, yearling sale and early 2‐year‐old racing, which could influence both catalogue placement and sales price.

The FFs represent offspring from mares that are unproven in terms of their ability to produce successful progeny at the time of sale. However, despite this, it appeared that buyers may be prepared to pay a premium for such individuals (Table 3). This is perhaps, because, if the dam goes on to produce other successful progeny, the value of their purchase will rise without the need for it to be successful itself. This finding is of particular interest to breeders, as it suggests that investments in stallion covering fee may be justified in mares that have not previously been bred (maiden mares).

In the present study, dams’ previous racing performance (DRW and DBT), when controlling for other attributes and interactions, had no association with sales price, with buyers instead appearing to value dams with proven progeny in terms of sibling race wins and sibling back type (SRW and SBT). In keeping with this, the only other study to evaluate yearling sales in the UK also demonstrated that if both dam and progeny had raced and won, then the progeny's race performance had a significantly greater influence on sales price than that of the dam.^11^ Progeny race performance attributes have consistently been positively associated with yearling sales price in previous studies.^6^, ^7^, ^11^, ^18^, ^19^, ^20^, ^23^ The present study, however, provides additional novel detail on this established relationship, with modelling of interactions demonstrating that the effect of SRW is significant only in yearlings from cheaper sires.

When all other interactions and attributes were accounted for, yearlings from larger consignments had significantly lower sales prices compared to those from smaller consignments, which could perhaps reflect adverse selection.^9^ For example, it is perceived that smaller consignments represent vendors selling a few, select high‐quality individuals, whereas large consignments represent vendors selling all stock, regardless of its quality, such as large commercial consignment operations compared to the often smaller, ‘owner/breeder’ type operations.

The main limitation of this study was the use of data from only one sale, which represented around one‐third of the UK foal crop and was less likely to include animals bred for national hunt (jump) racing and/or produced by owner/breeder enterprises, which may affect the generalisability of the findings. The pseudo *R* ^2^ = 0.64 also suggested that there could be unmeasured determinants that affect sales price, which are most likely to include physical attributes and measures of musculoskeletal health, given the need for athletic performance in this population. Such attributes can, however, be subjective and difficult to accurately quantify, whereas the present model, which utilises publicly available catalogue and stud book data, can more easily be applied by stakeholders.

## AUTHOR CONTRIBUTIONS

*Conception and design of the study*: Rebecca R. Mouncey, Pablo Alarcon and Kristien L. Verheyen. *Acquisition of the data*: Rebecca R. Mouncey. *Analysis of the data*: Rebecca R. Mouncey. *Interpretation of the data*: Rebecca R. Mouncey, Pablo Alarcon and Kristien L. Verheyen. *Drafting the article*: Rebecca R. Mouncey. *Critical revision for important intellectual content*: Rebecca R. Mouncey, Pablo Alarcon and Kristien L. Verheyen. *Final approval of the version to be published*: Rebecca R. Mouncey, Pablo Alarcon and Kristien L. Verheyen. *Accountability for all aspects of the work*: Rebecca R. Mouncey, Pablo Alarcon and Kristien L. Verheyen. The corresponding author confirms that she had full access to all the data in the study and takes responsibility for the integrity of the data and the accuracy of the data analysis.

## CONFLICTS OF INTEREST STATEMENT

The authors declare they have no conflicts of interest.

## ETHICS STATEMENT

Ethical approval was granted by the Royal Veterinary College's Social Sciences Research Ethical Review Board (URN SR2022‐ 0179).

## Supporting information

Supporting Information

## Data Availability

The data are publicly available from www.tattersalls.com/sales and www.racingpostbloodstock.com and Return of Mares—Weatherbys Shop.
