# Supplementary material for: Determinants of Thoroughbred yearling sales price in the UK
Source: Vet Rec Open. 2024 Jun 9;11(1):e81. doi: 10.1002/vro2.81 (PMC11162837; doi:10.1002/vro2.81)
Supplement: Supplementary file 1 — Supporting Information [file VRO2-11-e81-s001.pdf]

Supporting Information

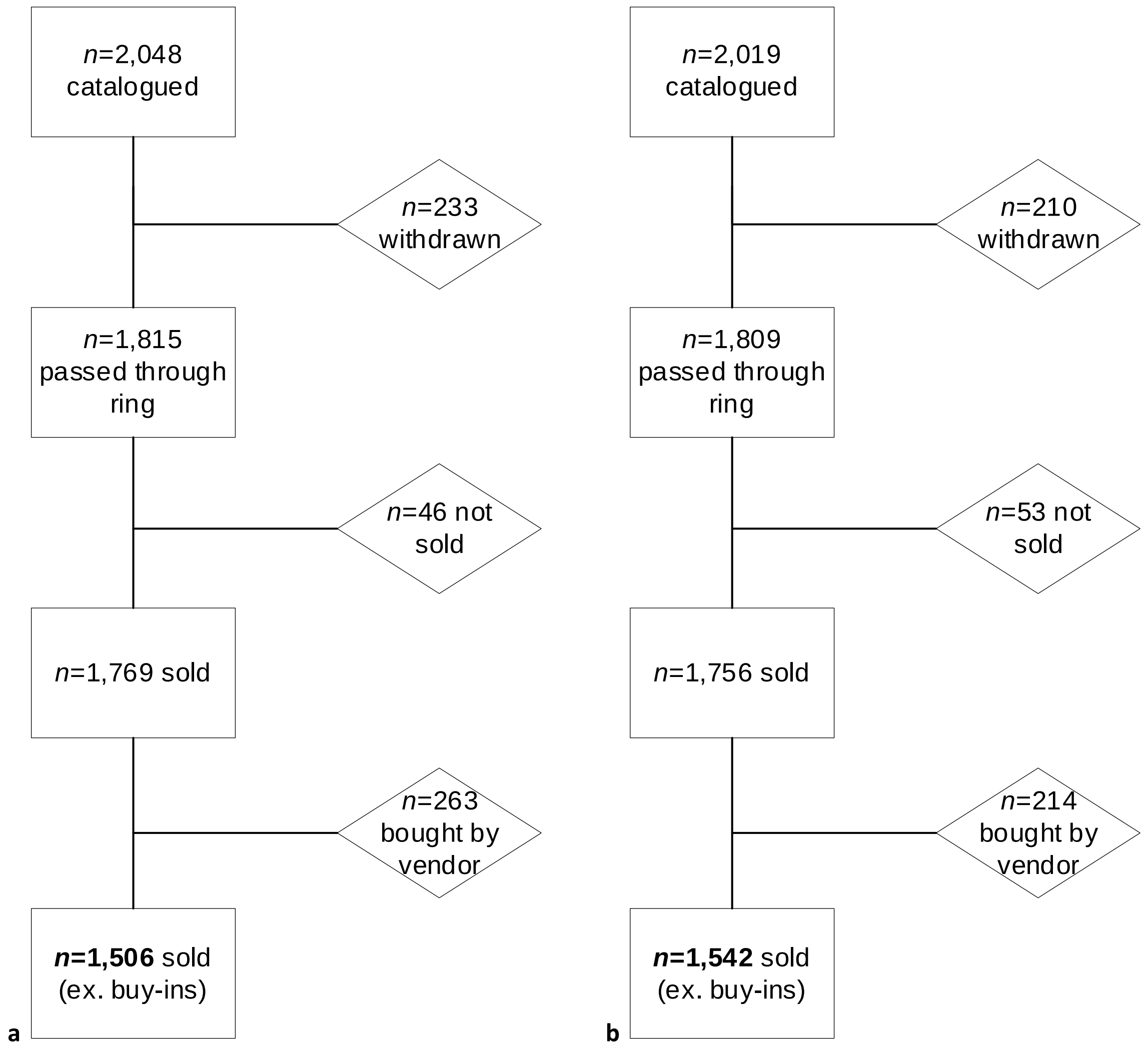

S1 Numbers of lots catalogued, passed through the ring and sold in the 2020 (a) and 2021 (b) Tattersalls October yearling sales

S2 Tattersalls sales house policies, the role of stallion covering fees and Weatherby's stud book registration

Tattersalls sales house dominates the British market, handling over 70% of all Thoroughbred yearlings sold in the UK, with the October yearling sale being the largest in Europe. The sale adopts the standard English ascending bid auction format in guineas (1.05 guineas = 1 GBP). An entry fee is payable by the vendor for each lot catalogued. All lots are subject to a reserve price, which is either the minimum selling price set by the auction or any higher price specified in advance by the vendor. The vendor must pay a commission of 5% of the sales price, whether sold through the ring or privately within 28 days of the sale, with an auction fee of 2.5% of the reserve price to inhibit the setting of unrealistic reserves. The auction fee is waived for lots selling for less than 20,000 guineas. Vendors are permitted to bid on their own lots and if the lot is bought by the vendor (bought-in), then the commission reduces to 2.5%.[1] The majority of yearlings sold at the Tattersalls October sale are bred for the purposes of flat racing, where horses cannot compete in a race until they are 2 years old, meaning that a yearling's actual ability, or lack thereof, remains unknown at the point of sale. A yearling's sales price therefore reflects both the individual's expected racing performance and its subsequent value as breeding stock, based primarily on its pedigree and physical attributes.

The Tattersalls October yearling sale is separated into four books.[2] Catalogue book placement is determined by the sales house, following examination of all yearlings by members of the sales team prior to the sale. Yearlings deemed to be of the highest quality and therefore expected to attain the highest prices, as judged both on pedigree and physical attributes, are placed in book 1. The yearling's placement within each book however and therefore, day of sale in books that run over multiple days (1, 2 and 3), is simply determined alphabetically by the first letter of the dam's registered name.

Pedigree information is provided in the sales catalogue[2] for three generations, alongside a summary of the dam lines' (1st, 2nd and 3rd dam) racing performance in terms of race wins, particularly 'black type'[3] performances (which are highlighted by the horse's name appearing in bold black type in the catalogue) of the dam herself and any siblings and/or progeny. Similarly, a summary of all featured stallions' (sires) race performance is provided alphabetically in a separate section of the catalogue.

The stallions' covering fee (the fee paid by the mare owner to the stallion owner following the successful covering of the mare with either the mare being confirmed in foal on the 1st of October following her last covering or following the birth of a live foal) is determined by the stallion owner and is set each year prior to the commencement of the breeding season in January. Fees go up or down in response to demand for coverings (the number of mares covered/season) and success of the stallion in terms of its progeny's race and breeding performance. The covering fee is therefore derived from a combination of the stallions' own race performance prior to going to stud, his progeny's race performance and, for older established stallions, his progeny themselves becoming successful breeding stock. First season sires are those that have not previously covered mares commercially and are therefore unproven in terms of progeny.

All Thoroughbred horses bred for the purpose of racing in the UK must be registered within 30 days of birth with Weatherbys Stud Book[4]. To be registered, both the sire and dam must also be Weatherbys registered and the covering (the breeding of the mare and stallion) must have been certified by the stallion owner to Weatherbys, as only foals conceived by natural covering (i.e. not artificial reproductive techniques) can be registered with the stud book authority. Weatherbys holds a central database of all coverings of registered mares and foals born in the UK, which is published annually in their "return of mares", available to purchase from their online shop[5].

---

[1] conditionsofsale.pdf (tattersalls.com)
[2] Tattersalls Newmarket
[3] 1st, 2nd or 3rd in a Group/Graded or Listed stakes race as approved by the Cataloguing Standards Guide available at: Submitted_By_2011_Part_I.qxd (tjcis.com)
[4] https://www.weatherbys.co.uk/general-stud-book/bloodstock-studbook
[5] Return of Mares 2023 – Weatherbys Shop

S3 Cataloguing distribution of lots sold (excluding buy-ins) in the 2020 Tattersalls October yearling sale

	Day			
**Book**	**1**	**2**	**3**	**Total**
**1**	123	119	126	368
**2**	216	213	208	637
**3**	237	222	0	459
**4**	42	0	0	42
**Total**	618	554	334	1,506

S4 Distribution of variables utilised to construct the hedonic price model to estimate Thoroughbred yearling sales price from all 1,506 sold lots (excluding buy-ins) from the 2020 Tattersalls October yearling sale

**Variable**	**Mean**	**Standard Deviation**	**Minimum**	**Maximum**
**Dependant variable**				
Ln (natural logarithm) Sales Price	10.55	1.43	6.73	15.09
**Sire attributes**				
Ln Stud Fee	9.97	0.92	7.89	13.02
FSS - First season sire	0.20	0.40	0	1
Coverings	123.33	51.56	1	228
**Dam attributes**				
DRW - dam race winner	0.61	0.49	0	1
DBT	0.25	0.43	0	1
SRW - sibling race winner	0.58	0.49	0	1
SBT - sibling back type	0.27	0.44	0	1
FF- first foal	0.15	0.36	0	1
Yearling attributes				
Colt	0.61	0.49	0	1
Age	574.46	30.81	493	649
**Sale attributes**				
Lots vendor	20.93	16.56	1	60
Book	2.12	0.80	1	4
Day	1.81	0.77	1	3

DBT = dam black type (1st 2nd 3rd in a Group/Graded or Listed stakes race as approved by the Cataloguing Standards Guide)

S5 Distribution of variables from the 2021 October yearling sale utilised to test the final model's forecasting ability

Variable	Mean	Standard Deviation	Minimum	Maximum
**Dependant variable**				
Ln (natural logarithm) Sales Price	10.82	1.34	6.73	14.27
Sire attributes				
Ln Stud Fee	10.09	0.98	7.46	13.07
FSS - First season sire	0.18	0.38	0	1
Coverings	102.66	61.90	1	216
**Dam attributes**				
DRW - dam race winner	0.62	0.48	0	1
DBT	0.25	0.43	0	1
SRW - sibling race winner	0.59	0.49	0	1
SBT - sibling back type	0.28	0.45	0	1
FF - first foal	0.14	0.35	0	1
**Yearling attributes**				
Colt	0.59	0.49	0	1
Age	570.46	31.09	494	650
**Sale attributes**				
Lots vendor	20.61	17.81	1	68
Book	2.12	0.81	1	4
Day	1.81	0.77	1	3

DBT = dam black type (1st 2nd 3rd in a Group/Graded or Listed stakes race as approved by the Cataloguing Standards Guide)

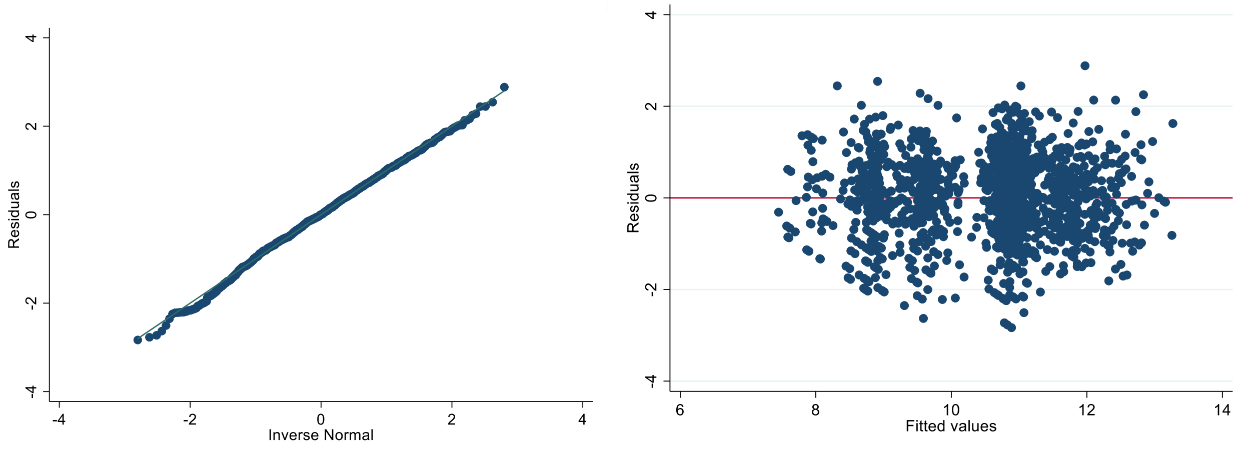

(a) (b)

S6 Testing (a) normality and (b) heteroscedasticity of residuals from the final model

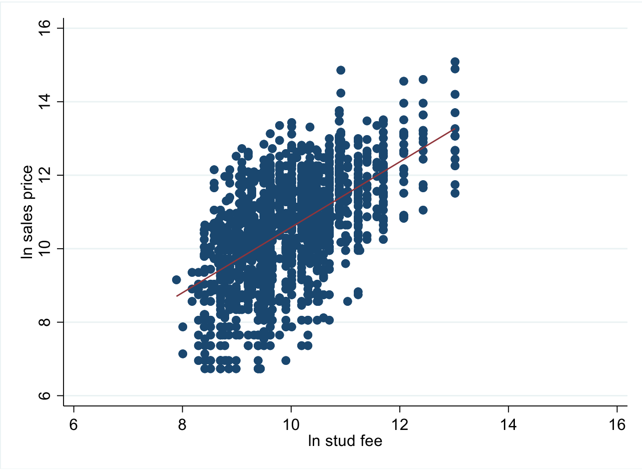

S7 Demonstrating linearity between the predictor (natural logarithm of stallion covering fee) and the outcome (natural logarithm of sales price)
